# Enhancing Bioethanol Productivity Using Alkali-Pretreated Empty Palm Fruit Bunch Fiber Hydrolysate

**DOI:** 10.1155/2018/5272935

**Published:** 2018-09-05

**Authors:** Seonghun Kim

**Affiliations:** Jeonbuk Branch Institute, Korea Research Institute of Bioscience and Biotechnology, 181 Ipsin-gil, Jeongeup 56212, Republic of Korea

## Abstract

Empty palm fruit bunch fiber (EPFBF) is a renewable resource in oil palm plantations that can be used for lignocellulosic bioethanol production. To enhance ethanol productivity with high-lignin-content EPFBF, the biomass was prepared with an alkali-thermal pretreatment (sodium hydroxide, 121°C, 60 min). The delignification yield was 55.4–56.9%, in proportion to the amount of sodium hydroxide, from 0.5 to 2.0 M. The lignin and hemicellulose contents of EPFBF were reduced by the pretreatment process, whereas the proportion of cellulose was increased. During enzymatic saccharification using Celluclast 1.5L and Novozyme 188 enzyme cocktails, about 62% of glucan was converted to a fermentable sugar. In simultaneous saccharification and fermentation, comparison among three ethanologenic yeast strains showed* Saccharomyces cerevisiae* W303-1A to be a candidate for maximum ethanol yield. In a batch fermentation with alkali-pretreated EPFBF hydrolysate, 21 g/L ethanol was obtained within 28 h, for a production yield of 0.102 g ethanol/g dry EPFBF or 0.458 g ethanol/g glucose. Moreover, a fed-batch fermentation produced 33.8±0.5 g/L ethanol with 1.57 g/L/h productivity in 20 h. These results show that the combination of alkaline pretreatment and biomass hydrolysate is useful for enhancing bioethanol productivity using delignified EPFBF.

## 1. Introduction

Lignocellulosic biomass is a renewable bioresource for second-generation bioethanol production. Lignocellulosic biomass is composed primarily of cellulose, hemicellulose, and lignin. Cellulose and hemicellulose can be hydrolyzed or degraded enzymatically to glucose and a variety of pentose and hexose sugars, respectively, which can then be fermented to produce bioethanol [1]. However, the rigid cellulose structure, combined with the amorphous hemicellulose and lignin cross-linked structure, is chemically complex and resistant to degradation [2]. Thus, physical, chemical, and biological methods are needed to convert the complex structures of lignocellulose into fermentable sugars [3, 4]. The production of bioethanol from lignocellulosic biomass requires four main steps: physical and chemical pretreatment of the lignocellulosic biomass, enzymatic hydrolysis of the lignocellulosic biomass, fermentation of the resulting sugars, and finally distillation of the ethanol [1]. Of these processes, pretreatment of the biomass is the most important step in saccharification efficiency, determining the ultimate bioethanol production yield [3, 5, 6]. The selection of the pretreatment procedure depends on the proportions of cellulose, hemicellulose, and lignin in the biomass and removing interfering materials from the biological steps that affect the overall bioprocess [3, 4].

The palm oil industries in Indonesia and Malaysia generate ~8.2 million tons of lignocellulosic agricultural waste and byproducts per year [7, 8]. An abundant byproduct in the palm oil industry is empty palm fruit bunch fiber (EPFBF), which consists of 27.6-32.5% lignin, 41.3-46.5% cellulose, and 25.3-33.8% hemicellulose [9, 10]. EPFBF contains a relatively higher lignin content rather than other agriculture residual biomasses and feedstocks as a comparison of per gram of biomass (% lignin: % cellulose: % hemicellulose); soybean straw (19.2:44.2:5.9); wheat straw (23.4:38.2:21.2); corn stover (17.6:37.5:22.4); and switch grass (17.6:31.0:20.4) [11–14]. Lignocellulosic EPFBF is potentially a low-cost material and an alternative renewable bioresource, instead of food sources, such as corn, sugar cane, and other food stocks, for the production of bioethanol [7, 8, 11].

EPFBF contains relatively high levels of hemicellulose and lignin per gram biomass, compared with other lignocellulosic biomasses [7–10]. Thus, pretreatment is needed to reduce the hemicellulose and lignin contents before bioethanol fermentation. The pretreatment processes should enhance the proportion of cellulose in the EPFBF [8–10]. Acidic, alkali, and sequential acidic-alkali pretreatments, combined with high temperature or high pressure, have been applied in “conventional” chemical treatment processes [3, 7, 12]. Acidic pretreatments are known to be effective with lignocellulosic biomasses in reducing the hemicellulose content. Similarly, alkali pretreatments have been reported as simple processes for the delignification of biomass under mild conditions with minimal sugar degradation and without the formation of inhibitory compounds [12]. Thus, both acid and alkali pretreatments can reduce hemicellulose and lignin together to enhance the cellulose content of a biomass [10, 13–15].

In this study, to increase the cellulose content of EPFBF, the alkali pretreatment was applied and the effects of this pretreatment were evaluated on reducing nonfermentable components in a lignocellulosic biomass for bioethanol production and productivity with the enzymatic biomass hydrolysate.

## 2. Methods

### 2.1. Materials

Empty palm fruit bunch fiber (EPFBF) was obtained from a local oil processing company in Malaysia. It was washed with tap water to remove soil and other particles and then dried at 105°C for 24 h. Celluclast 1.5L and Novozyme 188 were obtained from Novozymes Korea (Seoul, South Korea).

### 2.2. Chemical Pretreatment of EPFBF

Dried EPFBF (20% w/v) with no physical treatment was soaked in sodium hydroxide (NaOH) solution in the concentration range of 0.5–3 M and heated in an autoclave (121°C, 15 psi, 60 min). The physical reaction conditions were determined by pretest (Supplementary Table S1). The thermal-alkali-pretreated biomasses were removed from the black alkali solution and then washed with flowing tap water to remove NaOH from the biomass. This washing step was repeated several times until the washed water appeared a pale brown color. The alkali-pretreated EPFBF was dried at 105°C for 24 h and then kept under anhydrous conditions. The composition of the pretreated biomass was analyzed based on the NREL chemical analysis and testing laboratory analytical procedures (LAPs) of the US Department of Energy (DOE). The lignin content of the biomass was analyzed according to the LAPs of the DOE (LAP-003 and LAP-004). The biomass pretreatment with different NaOH concentration was performed in triplicate. The physical morphologies of the native and pretreated biomass materials were analyzed by scanning electron microscopy (SEM) using the previous method [13].

### 2.3. Enzymatic Hydrolysis of EPFBF

Enzymatic hydrolysis of the alkali-pretreated EPFBF (1 M NOH-treated biomass) was performed in a 50-mL cap-tube with a 10-mL working volume. EPFBF (10% w/v) was soaked in phosphate buffer (pH 6.0) and then the cellulase cocktails with the different concentration Celluclast 1.5L ranging from 20 to 100 filter paper units (FPU)) and the fixed concentration of Novozyme 188 (40 cellobiase units (CBU) of per gram cellulose) was added. The initial activities of Celluclast 1.5L and Novozyme 188 were 60 FPU per ml and 250 CBU per gram protein. 60 FPU of Celluclast 1.5L corresponds to 700 endoglucanase unit (EGU) per gram protein. One unit of endoglucanase activity corresponds to 1 *μ*mol of glucose released from carboxymethyl cellulose as the substrate per minute at 40°C and pH 6.0. One unit of cellobiase activity (CBU) is defined as 2 *μ*mole of glucose produced per minute at 40°C, pH 5. The enzymatic saccharification was carried out in a shaking incubator (42°C, 200 rpm, 72 h). Samples were withdrawn at each time point and then centrifuged (13,000 rpm, 10 min). The supernatant was removed and analyzed by HPLC to determine the amount of reducing sugars produced in the enzymatic reaction. All enzymatic saccharification of the biomass was performed in triplicate and repeated three times.

### 2.4. Microorganisms, Growth Conditions, and Cultivation.

*Saccharomyces cerevisiae* W303-1A [10, 13],* Kluyveromyces marxianus* CBS1555 [15, 16], and* Scheffersomyces stipitis* (*Pichia stipitis*) CBS5776 [17] were used as ethanol fermentation strains. Each yeast strain was cultivated in YPD medium (1% Bacto yeast extract, 2% Bacto peptone, and 2% glucose) at 30°C and 200 rpm for 24 h. For the batch flask cultures, 10% (w/v) EPFBF was prehydrolyzed with 100 FPU Celluclast 1.5L and 40 CBU Novozyme 188 cocktail for 12 h and then the 5% (v/v) seed culture with an initial optical density (OD600 nm) of ~5.0 was inoculated in the enzymatic saccharification solution containing 1% Bacto yeast extract and 2% Bacto peptone. Yeast cells were cultured further (30°C, 200 rpm, 24 h) for ethanol fermentation.

### 2.5. Batch and Fed-Batch Cultivation for Separated Saccharification and Fermentation (SHF)

For ethanol fermentation, 20% (w/v) alkali-pretreated EPFBF was hydrolyzed completely with 100 FPU Celluclast 1.5L and 40 CBU Novozyme 188 cocktail under the enzyme reaction conditions described above. After digestion, the enzymatic hydrolysate was centrifuged (20,000 rpm, 1 h) and the supernatant was filtered through a 0.22-*μ*m pore filter membrane system (Corning, Corning, NY, USA). For batch culture for SHF, 5% (v/v) preculture of* S. cerevisiae* strain was inoculated into a 1-L fermentor FMT ST-S (Fermentec, Cheongju, South Korea) with a 0.5 L working volume containing the filtered enzymatic hydrolysate of the biomass supplemented with 1% Bacto yeast extract and 2% Bacto peptone. The initial glucose concentration was 45.9 g/L. The fermentor was operated at 30°C with stirring at 200 rpm/min. For fed-batch cultivation, the filtrated enzymatic hydrolysate was added by step-feeding into the fermentor at the time point when the concentration of glucose was below 1 g/L. The other nutrient supplementation and the operation condition for the fed-batch fermentation except the feeding were the same to batch cultivation. The ethanol fermentation were performed in triplicate.

### 2.6. Analytical Procedures

Cell growth was monitored by measuring the optical density at 600 nm (OD600 nm) using a spectrophotometer. Total reducing sugars in the enzymatic saccharification reaction were measured using the 3,5-dinitrosalicylic acid method. The amounts of the released sugars in the enzymatic saccharification and the metabolites in fermentation were determined with a high-performance liquid chromatography (HPLC) system (Agilent Technologies, Santa Clara, CA, USA), equipped with a refractive index detector, an autosampler, and an Aminex HPX-87P column (7.8 × 300 mm; BioRad, Hercules, CA, USA) for monosaccharide analysis or a Rezex ROA-Organic Acid H^+^ column (7.8 × 300 mm; Phenomenex, Torrance, CA, USA) for organic acid analysis as described previously [10, 13–16]. All samples were clarified by filtration with a 0.20-*μ*m filter (Acrodisc LC PVDF Minispike; Pall Life Sciences, Ann Arbor, MI, USA) and then injected into the analytical HPLC column. The column temperature was kept at 65°C. The mobile phase was distilled water for monosaccharides and 2.5 mM sulfuric acid for organic acids, with a flow rate of 0.5 mL/min under isocratic conditions. All analyses were performed in triplicate.

## 3. Results and Discussion

### 3.1. Alkali Treatment of EPFBF and Its Composition

**Figure 1:**
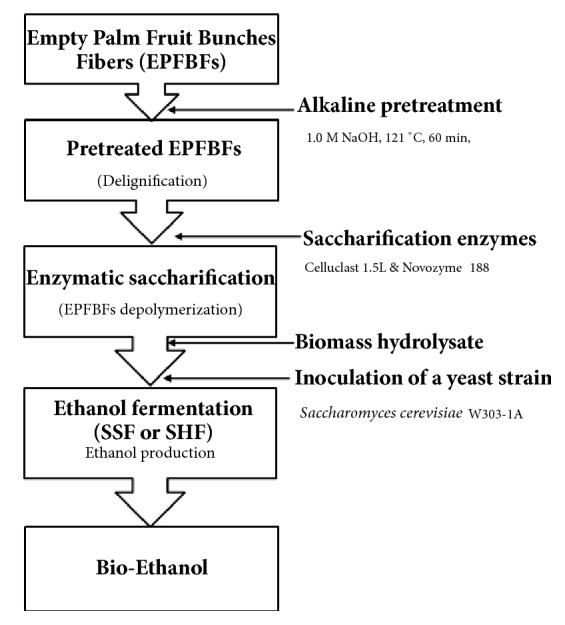
Overall scheme for bioethanol production from empty palm fruit bunch fiber (EPFBF).

To determine the composition of the EPFBF raw material prior to alkali pretreatment, the cellulose, hemicellulose, and lignin contents were analyzed using the analytical procedures of the US DOE. Dried EPFBF (100 g) consisted of 39% cellulose, 17% hemicellulose, and 28.8% lignin. Compared with previous data [7–9], the EPFBF used in this study contained a relatively high amount of lignin. Lignin interacts strongly with cellulose in biomass. It needs to be reduced or eliminated to enhance the enzymatic digestibility of the cellulose, to generate glucose, a fermentable sugar, and to increase bioethanol fermentation yield using yeast strains (Figure 1).

**Figure 2:**
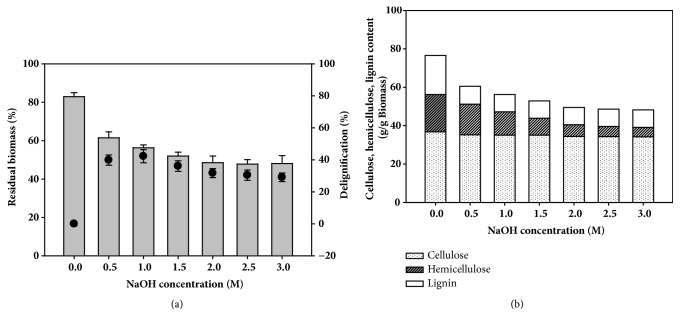
Composition of empty palm fruit bunch fiber (EPFBF) after pretreatment with different concentrations of NaOH at 121°C for 1 h. (a) Proportion of insoluble solids in alkali-pretreated EPFBF and delignification efficiency (close circles). (b) Contents of cellulose, hemicellulose, and lignin in EPFBF after NaOH pretreatment. All experiments were performed in triplicate.

To prepare a high-cellulose-content biomass, alkali pretreatment was performed to reduce lignin in the EPFBF. After the biomass had dried completely, the change in the chemical composition in alkali-pretreated EPFBF was analyzed (Figure 2). The concentration of sodium hydroxide affected the solubility of the biomass and the loss of cellulose, hemicellulose, and lignin contents (Supplementary Table S2). Alkali-thermal treatment within sodium hydroxide extracted up to 38.5–51.9% of the biomass into the soluble fraction. With 3 M sodium hydroxide pretreatment, the insoluble residue fraction contained 34.2 g cellulose, 4.9 g hemicellulose, and 9.0 g lignin per gram of the residual biomass. After alkali treatment, 10.2±0.5 % lignin and 18.9±1.2% hemicellulose were left in the residual biomass and the removal yields were ~71.1% and ~18.9%, respectively. Under high sodium hydroxide concentration from 2.5 M to 3.0 M, the half of the biomass was extracted as soluble fractions. On the other hand, the pretreated biomass did not lose much cellulose content, from 36.8 g to 34.2 g per gram of the residual EPFBF, with an increase in the sodium hydroxide concentration ranging from 0.5 to 2.0 M. The delignification yield with the alkali-thermal pretreatment was 55.4–56.9%, in proportion to the amount of sodium hydroxide. However, > 57% of the lignin could not be removed from the biomass under the alkali-thermal pretreatment conditions, even with a high concentration of sodium hydroxide. The alkali-thermal treatment of the biomass demonstrated that sodium hydroxide can extract hemicellulose and lignin effectively and increase the cellulose content per gram biomass.

Sodium hydroxide treatment was effective for delignification of the biomass. The main advantage is that the alkali process condition is relatively mild [12, 14, 18]. These mild conditions prevent condensation of lignin, resulting in a high lignin solubility. Due to the mild conditions, degradation of sugars to furfural, HMF, and organic acids, considering potential inhibitors for enzymatic saccharification as well as ethanol fermentation, is prevented. Moreover, the sodium hydroxide solution could be reused several times. In addition, to compare with other chemical pretreatment processes using aqueous ammonia, organosolv, and ionic liquids, the alkali treatment does not need the special equipment and the reactor. The cost of sodium hydroxide and of the alkali recovery is cheaper rather than the other chemical catalysts.

### 3.2. Enzymatic Hydrolysis of EPFBF

**Figure 3:**
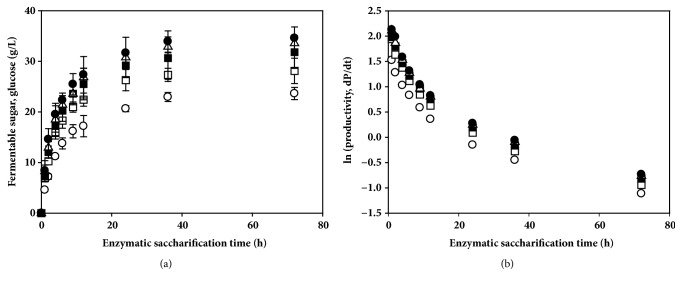
Effect of enzyme cocktail concentration on saccharification of alkali-pretreated empty palm fruit bunch fiber (EPFBF). (a) Fermentable sugar generation depended on the amount of Celluclast 1.5L/Novozyme 188 enzyme cocktail. (b) Sugar productivities of the enzyme cocktail ratio with the hydrolysis time: enzymatic saccharification was carried out at pH 4.8 and 42°C for 72 h. Then, 0.5 (open circle), 1.0 (open square), 1.5 (closed square), 2.0 (open triangle), and 2.5 FPU (closed circle) ratios of Celluclast 1.5L (FPU)/Novozyme 188 (CBU) were loaded per g dried EPFBF for enzymatic saccharification. All experiments were performed in triplicate. The data are expressed as mean ± SEM,* n* = 3.

To assess the enzymatic hydrolysis of alkali-thermal-pretreated EPFBF with 1 M sodium hydroxide, 10% (w/v) of the biomass, which contained 35.1% cellulose, 12.1% hemicellulose, and 8.8% lignin per 100 g dry biomass, was digested with different concentrations of cellulase (Celluclast 1.5L), from 20 to 100 FPU (filter paper units) and a constant concentration of *β*-glucosidase (Novozyme 188; 40 CBU) for 72 h. The amount of glucose generated by enzymatic saccharification increased with increasing amounts of cellulase per unit pretreated EPFBF (Figure 3(a)). At 72 h, the cellulose-degrading enzyme cocktail generated 23.6–34.6 g/L glucose and 14.1–14.6 g/L xylose. At enzyme loading ratios of 0.5 to 2.5 Celluclast 1.5L per Novozyme 188, the enzymatic digestibility efficiency at 72 h ranged from 42.3% to 62% with the pretreated biomass. At a ratio of 2.5 FPU/g cellulose to 1 CBU/g cellulose, 34.6 g/L glucose and 14.6 g/L xylose were obtained from the pretreated biomass. However, the untreated biomass saccharification with equal amount of cellulase cocktail dose produced 7.1–10.4 g/L glucose and 4.2–4.5 g/L xylose under equal enzyme dose (data not shown). Compared with untreated biomass, the alkali-thermal-pretreated EPFBF produced 2-3 times higher amounts of fermentable sugars in the enzymatic hydrolysis. The chemical pretreatment could extract lignin from the biomass and reduce the strong interactions in cellulose-hemicellulose-lignin complexes in the biomass structure, enhancing the enzyme reaction efficiency [19]. Moreover, the morphological analysis of the untreated and the alkaline-treated biomass by scanning electron microscopy (SEM) showed clearly that the alkaline pretreatment made the biomass surface many cracks, porous, and rough structures. These morphological changes in the pretreated biomass might be for the cellulase to enhance accessibility as well as enzymatic hydrolysis of the polysaccharide structures (Supplementary Fig.  S1).

Nevertheless, an increase in enzyme dose did not significantly affect the amount of fermentable sugar generation. At the final reaction time point, the amount of glucose increased up to a 1.5 enzyme loading ratio of Celluclast®1.5/Novozyme 188, whereas the sugar concentrations decreased at 2.0 and 2.5 enzyme cocktail ratios (data not shown). It would be a product inhibition for the cellulase: the monosaccharides, disaccharides, and oligosaccharides generated by enzymatic hydrolysis might inhibit cellulase cocktails as a product inhibition. High concentration sugars produced by the cellulase could decrease further hydrolysis reaction [20].

Additionally, sugar production increased time-dependently, whereas enzymatic hydrolysis rates decreased exponentially due to product inhibition (Figure 3(b)). The generated glucose, xylose, and incompletely digested cellobiose may inhibit the hydrolysis reactions of the cellulase cocktail enzymes.

The amount of xylose hydrolyzed from xylan in the biomass showed a near-constant value of ~14 g/L, even when the enzyme loading ratio was changed. The constant amount of xylose with enzyme supplementation was probably due to the *β*-xylosidase activity present in Novozyme188, which was loaded at a fixed concentration (40 CBU). All of the data presented for the enzymatic saccharification showed that pretreated EPFBF could be used to generate high concentrations of glucose, rather than xylose and other sugars, for bioethanol production.

### 3.3. Simultaneous Saccharification and Fermentation of Alkali-Pretreated EPFBF

To test ethanol production with the enzymatic hydrolysis of alkali-pretreated EPFBF, three yeast strains,* Saccharomyces cerevisiae* W303-1A [10, 13],* Kluyveromyces marxianus* CBS1555 [15, 16], and* Scheffersomyces stipitis* (*Pichia stipitis*) CBS5776 [17], were used as ethanol producers. Batch cultivation of each yeast strain was performed in a 50-mL culture volume in a 250-mL Erlenmeyer flask with 5 g of alkali-pretreated EPFBF, supplemented with a 100 FPU Celluclast 1.5L and 40 CBU Novozyme 188 cocktail. Before cell inoculation, prehydrolysis was performed at 42°C for 12 h. Then, 5% (v/v) yeast inoculum (5 OD600 nm) was added and cultured further (30°C, 200 rpm, 18 h) for ethanol fermentation. The prehydrolysis step generated ~12.3±1.5 g/L glucose in each flask for yeast cell growth with no lag phase. The ethanol productivity of each yeast strain under these culture conditions is summarized in Table 1.

With simultaneous saccharification and fermentation (SSF),* S. cerevisiae* W303-1A was the “best” strain for maximum ethanol concentration and production yield: 10% (w/v) alkali-pretreated EPFBF was converted to 14.5 g/L ethanol with 0.14 g ethanol/g dried biomass. The yeast used 82.2% of the fermentable sugars for ethanol production, and no reduction in ethanol concentration was observed in culturing for 18 h. On the other hand,* K. marxianus* CBS1555 and* Scheffersomyces stipitis* CBS5776 produced 10.6 g/L ethanol with 0.11 g ethanol/g dried biomass and 10.1 g/L ethanol with 0.10 ethanol/g dried biomass, respectively. These yeast strains converted 60.1% and 57.2% glucose, respectively, in the biomass hydrolysate to ethanol.

In SSF, among the yeast strains tested,* S. cerevisiae* W303-1A had maximum ethanol productivity and yield (Table 1). Although inoculum size and initial glucose concentration were almost equal amounts in culture broth, the ethanol productivity of* S. cerevisiae *W303-1A was about 1.4-fold higher than those of* K. marxianus* CBS1555 and* Scheffersomyces stipitis* CBS5776 strains. These different productivities among the ethanologenic yeast might be due to the individual strain characteristics such as glucose uptake and metabolism, stress responsibility, and redox balance in the biomass hydrolysate [21].

### 3.4. Batch Fermentation Supplemented with Alkali-Pretreated EPFBF Hydrolysate

**Figure 4:**
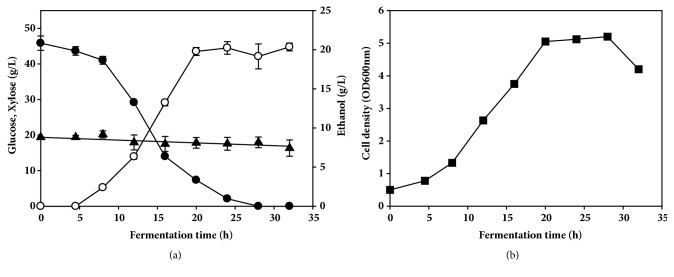
Batch fermentation of enzymatically hydrolyzed EPFBF by* S. cerevisiae* W303-1A in a 1 L jar fermentor. (a) The amounts of glucose (closed circle), xylose (closed triangle), and ethanol (open circle) were analyzed by HPLC. (b) Cell growth (closed square) was monitored by measuring the absorbance at 600 nm using a spectrophotometer. All analyses were performed in triplicate. The data are expressed as mean ± SEM,* n* = 3.

To assess the fermentability of the alkali-pretreated EPFBF hydrolysate, separate hydrolysis and fermentation reactions were performed using* S. cerevisiae* W303-1A in a 1 L jar fermentor. The enzymatic hydrolysate was prepared as described in Methods. The initial concentrations of glucose and xylose in the hydrolysate of alkali-pretreated EPFBF were 47.4±2.5 g/L and 19.0±1.2 g/L, respectively. After yeast inoculation, ethanol fermentation proceeded gradually for 30 h (Figure 4(a)). Yeast cell growth was slow until 4.5 h in a lag phase, increased exponentially, and entered a stationary phase between 16 and 24 h (Figure 4(b)). The concentration of glucose decreased gradually due to cell growth. All of the glucose in the culture broth was depleted at 28 h. The amount of xylose, which is a nonfermentable sugar, did not decrease.

The highest ethanol concentration was 21 g/L at 28 h, giving an ethanol yield of 0.102 g ethanol/g dry EPFBF and 0.458 g ethanol/g glucose at 28 h. Ultimately, 85.4% of the fermentable sugar was used by the yeast for ethanol fermentation. No reduction in ethanol concentration was observed until glucose depletion. Cell growth and ethanol production in the separate hydrolysis and fermentation (SHF) were faster than those in the simultaneous saccharification and fermentation (SSF) (Supplementary Fig.  S2). Ethanol fermentation of the alkali-pretreated EPFBF by* S. cerevisiae* W303-1A produced several metabolites, including acetic acid, lactic acid, succinic acid, and glycerol (data not shown). All metabolites produced by the yeast were at concentrations > 1 g/L, lower than that of the ethanol. In addition, these metabolites accumulated with no consumption in the fermentation broth. These metabolites did not appear to affect cell growth or ethanol productivity during the fermentation. Levels of these byproduct metabolites were probably too low to have any negative effects on cell metabolism due to the short fermentation time in the SHF process. These results indicated that SHF produced large amounts of ethanol with less byproducts and no inhibition by metabolites in the short fermentation time.

### 3.5. Fed-Batch Fermentation Supplemented with Alkali-Pretreated EPFBF Hydrolysate

**Figure 5:**
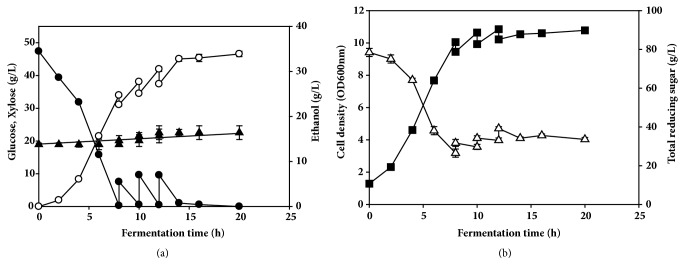
Fed-batch separate hydrolysis and fermentation using the alkali-pretreated EPFBF hydrolysate with* S. cerevisiae* W303-1A in a 1 L jar fermentor. (a) The amounts of glucose (closed circle), xylose (closed triangle), and ethanol (open circle) were analyzed by HPLC. (b) Cell growth (closed square) was monitored by measuring the absorbance at 600 nm using a spectrophotometer. Total reducing sugar (open triangle) was measured using the 3,5-dinitrosalicylic acid method. All analyses were performed in triplicate. The data are expressed as mean ± SEM,* n* = 3.

To increase ethanol production and yield, separate hydrolysis and fermentation in a fed-batch were performed using the alkali-pretreated EPFBF hydrolysate. When the residual glucose concentration fell below 0.5 g/L during the fermentation, the filtered enzymatic hydrolysate was fed into the fermentor to maintain ~10.0±1.5 g/L glucose. By 20 h, 33.8±0.5 g/L ethanol was produced (Figure 5(a)), and the residual glucose concentration was completely depleted. The productivity of ethanol was 1.57 g/L/h, and the production yield was 0.102 g per g EPFBF and 0.465 g per g glucose, with 91.2% sugar conversion efficiency.

The yeast strain grew exponentially with no lag phase for 4 h and then entered a stationary phase at 8 h (Figure 5(b)). The cell density was maintained until the culture end time with no growth decrease. The initial glucose was consumed rapidly by the yeast by 8 h. In addition, the fed sugar in each 2-h period was also taken up rapidly by the viable yeast cells until the fermentation end point. Xylose could not be fermented by the yeast and thus accumulated in the fermentor (Figure 5(a)). Total reducing sugar decreased markedly until 8 h in the fermentation, and then the nonfermentable sugar increased gradually (Figure 5(b)).

In the fed-batch cultivation, byproduct metabolites, such as organic acids and glycerol, were produced, but at < 0.53±0.12 g/L by the end of fermentation (data not shown). The cultivation time for ethanol production was only 20 h until the glucose was completely depleted. It may be that the byproducts did not accumulate in the fed-batch culture due to the short fermentation time. These results showed that separate hydrolysis and fermentation in a fed-batch using the alkali-pretreated EPFBF hydrolysate could produce bioethanol with high productivity in a short operation time.

## 4. Conclusions

Alkali-thermal pretreatment of EPFBF with sodium hydroxide was effective in reducing hemicellulose and lignin contents and enhancing the enzymatic digestibility and fermentability of the biomass. The alkali-pretreated EPFBF showed ~55.4% delignification efficiency. In the pretreated biomass, 62% of the cellulose was hydrolyzed by the Celluclast 1.5L/Novozyme 188 enzyme cocktail and was converted to fermentable sugars during enzymatic saccharification. In a small-scale culture of simultaneous saccharification and fermentation supplemented with the pretreated biomass,* S. cerevisiae*, the best ethanol producer among the three yeast strains tested, produced 14.5 g/L ethanol with 0.14 g ethanol/g biomass and 82.2% fermentable sugar conversion. In a batch involving separate hydrolysis and fermentation supplemented with the alkali-pretreated EPFBF hydrolysate, 21 g/L ethanol was obtained within 28 h for a production yield of 0.102 g ethanol/g dry EPFBF or 0.458 g ethanol/g glucose. In addition, a fed-batch involving separate hydrolysis and fermentation could produce 33.8±0.5 g/L ethanol with 1.57 g/L/h productivity in only 20 h. These results confirm that alkali-pretreated EPFBF effectively reduced the hemicellulose and lignin components. Moreover, separate hydrolysis and fermentation using biomass hydrolysate may be useful for producing bioethanol with high productivity.

## Figures and Tables

**Table 1:** Comparison of ethanol production among three yeast strains.

Yeast strain	*Saccharomyces cerevisiae* W303-1A	*Kluyveromyces marxianus* CBS1555	*Scheffersomyces stipites* CBS5776
Max. ethanol (g/L)	14.5±0.2	10.6±0.1	10.1±0.2
Ethanol yield (EtOH g/ g EPFBFs)	0.14±0.01	0.11±0.01	0.10±0.01
Ethanol productivity (g/L/h)	0.81±0.01	0.59±0.01	0.56±0.01
Inoculation (OD_600nm_)	5.3±0.1	5.2±0.1	5.4±0.1
Initial glucose (g/L)	12.3±1.5	12.3±1.5	12.3±1.5
Residual xylose (g/L)	7.3±0.1	6.2±0.1	5.4±0.1

## Data Availability

The data used to support the findings of this study are available from the corresponding author upon request.

## Supplementary Materials

Supplementary Materials
**Table S1**. Comparison of the contents of cellulose, hemicellulose, and lignin in the thermal-alkaline-pretreated EPFBFs under different temperatures (the alkaline pretreatment of EPFBFs was performed with 0.5 M NaOH at each temperature for 60 min).** Table S2.** The composition of cellulose, hemicellulose, and lignin in the EPFBF biomass before and after the treatment.** Fig. S1.** Scanning electron microscopy (SEM) analysis of the EPFBF samples, the native EPFBF** (A),** and the alkaline-treated EPFBF (B), respectively.** Fig. S2.** Batch culture of simultaneous saccharification fermentation (SSF) of the alkaline-pretreated EPFBF by* S. cerevisiae* W303-1A in a 1 L jar fermentor. A total of 0.5 L of working volume containing 10% (w/v) of the pretreated biomass was used. The fermentation was operated at 30°C with stirring at 200 rpm/min. Glucose, xylose, and ethanol profiles in the fermentor: filled circles, glucose; filled triangles, xylose; open squares, ethanol.
