# Supplementary material for: Enhancing Bioethanol Productivity Using Alkali-Pretreated Empty Palm Fruit Bunch Fiber Hydrolysate
Source: Biomed Res Int. 2018 Sep 5;2018:5272935. doi: 10.1155/2018/5272935 (PMC6145314; doi:10.1155/2018/5272935)
Supplement: Supplementary Materials — Table S1. Comparison of the contents of cellulose, hemicellulose, and lignin in the thermal-alkaline-pretreated EPFBFs under different temperatures (the alkaline pretreatment of EPFBFs was performed with 0.5 M NaOH at each temperature for 60 min). Table S2. The composition of cellulose, hemicellulose, and lignin in the EPFBF biomass before and after the treatment. Fig. S1. Scanning electron microscopy (SEM) analysis of the EPFBF samples, the native EPFBF (A), and the alkaline-treated EPFBF (B), respectively. Fig. S2. Batch culture of simultaneous saccharification fermentation (SSF) of the alkaline-pretreated EPFBF by S. cerevisiae W303-1A in a 1 L jar fermentor. A total of 0.5 L of working volume containing 10% (w/v) of the pretreated biomass was used. The fermentation was operated at 30°C with stirring at 200 rpm/min. Glucose, xylose, and ethanol profiles in the fermentor: filled circles, glucose; filled triangles, xylose; open squares, ethanol. [file 5272935.f1.docx]

**Supplementary materials**

**Table S1.** Comparison of the contents of cellulose, hemicellulose, and lignin in the thermal-alkaline-pretreated EPFBFs under different temperatures^1)^

| Temperature  [°C] | Residual biomass  [g] | Cellulose  [g/g] | Hemicellulose  [g/g] | Lignin  [g/g] |
| --- | --- | --- | --- | --- |
| Untreated biomass | 100.0 | 36.3±0.5 | 19.7±1.6 | 30.6±1.2 |
| 121 | 61.5 | 30.8±1.2 | 16.0±1.1 | 9.3±0.5 |
| 150 | 53.0 | 31.6±0.7 | 14.9±0.3 | 8.6±0.4 |
| 160 | 48.5 | 34.4±0.5 | 16.7±0.6 | 8.2±0.5 |
| 170 | 46.2 | 37.5±0.9 | 17.8±1.2 | 7.9±0.6 |
| 180 | 43.7 | 36.6±0.4 | 16.7±1.0 | 7.6±0.4 |

^1)^ The alkaline-pretreatment of EPFEFs were performed with 0.5 M NaOH at each temperature for 60 min.

**Table S2.** The composition of cellulose, hemicellulose, and lignin in the EPFBF biomass before and after the treatment

| NaOH  [M] | Cellulose  [g] | Hemicellulose  [g] | Lignin  [g] |
| --- | --- | --- | --- |
| Untreated biomass | 36.3±0.5 | 19.7±1.6 | 30.6±1.2 |
| 0.0 | 36.8±1.2 | 19.4±1.1 | 20.4±0.5 |
| 0.5 | 35.2±0.7 | 16.0±0.8 | 9.3±0.3 |
| 1.0 | 35.1±0.5 | 12.1±0.6 | 9.0±0.1 |
| 1.5 | 35.1±0.9 | 8.8±0.7 | 9.0±0.4 |
| 2.0 | 34.4±0.5 | 6.1±0.5 | 9.0±0.5 |
| 2.5 | 34.3±0.3 | 5.3±0.6 | 9.0±0.4 |
| 3.0 | 34.2±0.4 | 4.9±0.5 | 9.0±0.3 |

**
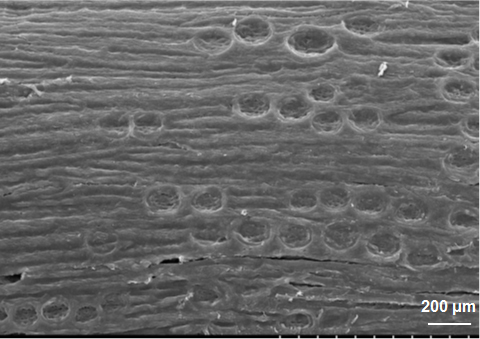
(A)**

**
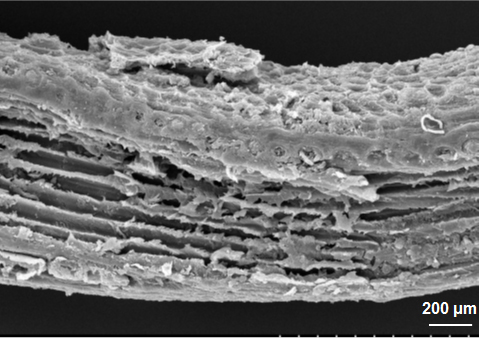
(B)**

**Fig. S1.** Scanning electron microscopy (SEM) analysis of the EPFBF samples, the native EPFBF (**A**) and the alkaline-treated EPFBF (B), respectively.

**Fig. S2.** Batch culture of simultaneous saccharification fermentation (SSF) of the alkaline-pretreated EPFBF by S. cerevisiae W303-1A in a 1 L-jar fermentor. A total of 0.5 L of working volume containing 10% (w/v) of the pretreated biomass was used. The fermentation was operated at 30°C with stirring at 200 rpm/min. Glucose, Xylose, and Ethanol profiles in the fermentor: filled circles, glucose; filled triangles, xylose; open squares, ethanol.
